# Thioridazine as Chemotherapy for Mycobacterium avium Complex Diseases

**DOI:** 10.1128/AAC.02985-15

**Published:** 2016-07-22

**Authors:** Devyani Deshpande, Shashikant Srivastava, Sandirai Musuka, Tawanda Gumbo

**Affiliations:** a Center for Infectious Diseases Research and Experimental Therapeutics, Baylor Research Institute, Baylor University Medical Center, Dallas, Texas, USA; b Department of Medicine, University of Cape Town, Observatory, South Africa

## Abstract

Mycobacterium avium-intracellulare complex (MAC) causes an intractable intracellular infection that presents as chronic pulmonary disease. Currently, therapy consists of ethambutol and macrolides and takes several years to complete. The neuroleptic phenothiazine thioridazine kills mycobacteria by inhibiting the electron transport chain. In several experiments with bacterial populations of up to 10^12^ CFU/ml, we failed to isolate any bacteria resistant to 3 times the MIC of thioridazine, suggesting the absence of resistant mutants at bacterial burdens severalfold higher than those encountered in patients. In the hollow-fiber model of intracellular MAC (HFS-MAC), thioridazine achieved an extracellular half-life of 16.8 h and an intracellular half-life of 19.7 h. Thioridazine concentrations were >28,000-fold higher inside infected macrophages than in the HFS-MAC central compartment (equivalent to plasma). Thioridazine maximal kill was 5.20 ± 0.75 log_10_ CFU/ml on day 7 (*r*^2^ = 0.96) and 7.19 ± 0.31 log_10_ CFU/ml on day 14 (*r*^2^ = 0.99), the highest seen with any drug in the system. Dose fractionation studies revealed that thioridazine efficacy and acquired drug resistance were driven by the peak concentation-to-MIC ratio, with a 50% effective concentration (EC_50_) of 2.78 ± 0.44 for microbial killing. Acquired drug resistance was encountered by day 21 with suboptimal doses, demonstrating that fluctuating drug concentrations drive evolution faster than static concentrations in mutation frequency studies. However, the thioridazine EC_50_ changed 16.14-fold when the concentration of fetal bovine serum was changed from 0% to 50%, suggesting that intracellular potency could be heavily curtailed by protein binding. Efficacy in patients will depend on the balance between trapping of the drug in the pulmonary system and the massive intracellular concentrations versus very high protein binding of thioridazine.

## INTRODUCTION

*M*ycobacterium avium complex (MAC) infection leads to relentless chronic pulmonary disease in immunocompetent people and disseminated disease in immunocompromised patients. In the United States, the incidence of pulmonary MAC now surpasses that of tuberculosis, making it the most important mycobacterial infection in North America (1, 2). Both disseminated and pulmonary diseases are predominantly intracellular. In pulmonary MAC, immunohistochemistry has revealed intracellular MAC in monocyte-lineage cells in alveoli and infected multinucleated giant cells in necrotic lesions (3). The bacterial burden in the lung is unclear but likely is similar to that of Mycobacterium tuberculosis in cavitary pulmonary disease at about 10^6^ CFU/ml. In disseminated disease, the burden of organisms in the blood is variable, ranging from 1 to 10^4^ CFU/ml (4, 5). Current therapy consists of a backbone of macrolide and ethambutol, with the occasional addition of a rifamycin. Microbiologic success is achieved in roughly half of the patients treated for disseminated MAC, unless patients achieve immune reconstitution. In patients with pulmonary MAC, microbial response rates are also poor, and therapy duration is several years. Given that the treatment for both pulmonary and disseminated disease lasts at least a year and microbial response rates are poor, it is imperative to design therapy regimens with agents that achieve high intracellular levels and rapidly kill MAC.

Thioridazine, a phenothiazine derivative, has been shown to have efflux pump inhibiting activity *in vitro* and *ex vivo* (6–8). In the past, we have demonstrated that thioridazine has excellent efficacy against all 3 metabolic populations of the extracellular M. tuberculosis predominant in cavitary pneumonia (9). However, it achieved optimal microbial killing at concentrations that we considered toxic to tuberculosis (TB) patients. Nevertheless, given that thioridazine kills M. tuberculosis and could have high intracellular concentrations in the lungs because of lysosomal trapping (10), we were interested in determining if it had efficacy against the more intracellular MAC. We were interested in identifying the pharmacokinetics/pharmacodynamics (PK/PD) of thioridazine against this intracellular pathogen in order to help design optimal doses and dose schedules in the future and, if possible, look at congeners. Therefore, we tested its efficacy using a PK/PD design in our hollow-fiber model of disseminated MAC (HFS-MAC) (11–14).

## MATERIALS AND METHODS

### Microorganisms and chemicals.

MAC (ATCC 700898) stock cultures were stored in Middlebrook 7H9 broth and 10% glycerol at −80°C. Bacterial stock was thawed and incubated at 37°C in a water bath in Middlebrook 7H9 broth with 10% oleic acid-albumin-dextrose-catalase (OADC) under shaking conditions for 4 days to achieve exponential-phase growth for each experiment. Methicillin-resistant Staphylococcus aureus (MRSA) (ATCC 29213) was cultured in Mueller-Hinton broth at 37°C overnight for the protein binding studies. Hollow-fiber cartridges were purchased from FiberCell (Frederick, MD). RPMI 1640 was purchased from Sigma (St. Louis, MO), and fetal bovine serum (FBS) was purchased from SAFC Biosciences (St. Louis, MO) and Sigma. FBS was heat inactivated and filtered prior to use. Thioridazine hydrochloride in powder form was obtained from Sigma. The drug was serially diluted using distilled water to the drug concentrations required for study. Throughout all experiments, thioridazine was shielded from direct light.

### Assay to identify effect of thioridazine protein binding on microbial kill.

In order to determine the effect of the extent of protein binding on thioridazine's effect on microbial killing, we cultured MRSA in non-cation-adjusted Mueller-Hinton broth to attain exponential-phase growth to a turbidity of a 0.5 McFarland standard. Triplicates of the culture were then inoculated into 3 sets of prewarmed RMPI with either 0% FBS, 2% FBS, or 10% FBS to achieve a final desired bacterial burden of 10^5^ CFU/ml. Cultures were coincubated with thioridazine at concentrations of 0, 10, 20, 30, 40, 50, 80, and 100 mg/liter for 6 h under vigorous shaking conditions at 37°C. Following incubation, cultures were serially diluted and spread on Mueller-Hinton agar. The cultures were then incubated for 36 h at 37°C and CFU were counted. Protein binding was calculated as the percent change in the potency of thioridazine. Potency is defined as the concentration of thioridazine mediating 50% of maximal killing (EC_50_).

We also wanted to determine the effect of thioridazine protein binding on intracellular infection. Human-derived THP-1 macrophages (ATCC TIB-202) were cultured in prewarmed RPMI 1640 medium and 10% FBS. The THP-1 cells were activated using 10^−3^ μM phorbol myristate ester for 72 h in 12-well plates until they were adherent, as described in the past (11–14). The adherent cells, which were 1 × 10^6^ THP-1 cells/ml, then were infected with 5 × 10^4^ CFU/ml of MRSA in log-phase growth, which was added to the wells to reach a multiplicity of infection of 1 bacteria to 20 macrophages. After incubation for 1 h at 35°C under 5% CO_2_, bacteria were washed off twice with warm RPMI 1640 with streptomycin. Thioridazine in RPMI 1640 plus one of four different amounts of FBS then was added to the wells, making final concentrations as high as 32 mg/liter and 2-fold dilutions down to 0.25 mg/liter in triplicate. The amounts of FBS in RPMI that were tested were 0%, 2%, 10%, and 50%. The cultures then were incubated for 2 h at 35°C under 5% CO_2_, after which the macrophages were examined under a microscope. The cells were then washed twice in warm RMPI, ruptured using 0.5% Triton X-100, and then serially diluted in saline and cultured overnight on Muller-Hinton agar at 35°C under 5% CO_2_ for colony counts. The relationship between microbial effect and thioridazine was performed using an inhibitory sigmoid maximal kill (*E*_max_) model, and the EC_50_s under the different percentages of FBS were compared.

### Determination of MIC and mutation frequency.

The thioridazine MIC for MAC was determined using the agar dilution method on two different occasions. Mutation frequencies to 3 times the MIC were also determined on agar supplemented with thioridazine. Twenty-four sets of agar each were inoculated with 200 μl of exponential-growth-phase MAC at the following bacterial burdens of inoculum: 8.0 log_10_ CFU, 9.0 log_10_ CFU, 11.0 log_10_ CFU, and 12.0 log_10_ CFU. The cultures were incubated for 14 days at 37°C, after which colonies were counted. The lower limit of detection of MAC colonies in this assay was 0.3 log_10_ CFU/ml.

### Exposure effect study of extracellular MAC.

In order to study the activity of thioridazine against extracellular MAC, bacteria on day 4 of log-phase growth were adjusted to a bacterial density of 1.5 × 10^6^ CFU/ml. Bacterial suspension in broth was treated with thioridazine at concentrations of 10, 20, 25, 30, 40, 50, and 60 mg/liter for 7 days in 24-well plates. Cultures were washed, serially diluted, spread on agar, and incubated at 37°C under 5% CO_2_ for 14 days, and then colonies were counted.

### Thioridazine exposure-effect of intracellular MAC.

THP-1 macrophages were cultured as described above in RPMI 1640 medium plus 10% FBS. A culture of 1.5 × 10^5^ CFU/ml was used to infect 1.5 × 10^6^ macrophages/ml by coincubating overnight at 37°C under 5% CO_2_, giving a bacillus-to-macrophage multiplicity of infection of 1:10. The infected macrophages were washed twice with warm RPMI 1640 and 10% FBS by centrifugation at 100 × *g* for 5 min and then examined in a hemocytometer for cell counts and viability after staining with trypan blue. This resulted in an intracellular infection with a bacillary burden of 3.75 log_10_ CFU/ml. The infected cells were treated in triplicate with 10, 20, 25, 30, 40, 50, and 60 mg/liter of thioridazine in 24-well plates for 7 days. Cell lysis was performed using 0.5% Triton X-100, and lysate was plated on agar and incubated for 14 days at 37°C under 5% CO_2_.

### Dose-effect and dose fractionation studies in the HFS-MAC.

The HFS-MAC has been described in detail in the past (11, 12, 14). The system is used to mimic the human concentration-time profiles of antibiotics in the central and peripheral compartments, which circulate media but are cell free. We inoculated 20 ml of MAC-infected THP-1 monocytes into each hollow-fiber system. In a pilot study we used RPMI with 10% FBS as the circulating media. The study failed (see Results); therefore, we performed several studies to optimize the model. First, we performed studies with MRSA in RPMI with different percentages of FBS to delineate the role of protein binding described above. We then changed the percentage of FBS based on the MRSA study results. Second, we preconditioned hollow-fiber systems with once-daily thioridazine doses under the control of a computerized syringe pump, starting 3 days prior to inoculation of infected MAC. Third, thioridazine concentrations were changed 10-fold and targeted to achieve peak concentrations of 0, 3.5, 7, 10.5, 14, 17.5, 35, and 70 mg/liter, and thioridazine was administered daily for 14 days at a half-life of 17 h based on the drug's highly variable late disappearance half-life in patients after multiple doses (i.e., between 17 and 25 h) (15). In order to validate that intended concentration-time profiles had been achieved, the central compartment of each HFS-MAC unit was sampled at 0, 1, 5, 8, 16, and 24 h after the last dose of the experiment. We also simultaneously sampled the infected macrophages in the peripheral compartment of the HFS-MAC at each of these time points. The samples were then used to identify both extracellular and intracellular thioridazine pharmacokinetics. For bacterial burden and macrophage cell counts, the peripheral compartment was sampled as described for the pilot study on days 0, 3, 5, 7, and 14, and cultures were performed as described for the pilot study.

Dose fractionation studies next were performed using EC_20_, EC_50_, and EC_80_ exposures, identified in dose-effect studies based on recommendations for performance of PK/PD studies (16). This step was performed to break the colinearity of *C*_max_/MIC, area under the concentration-time curve (AUC)/MIC, and the percentage of time the concentration persists above the MIC (%*T*_MIC_). Each of the exposures was administered using one of three dose schedules: single dose once a week, the single dose divided equally into two doses and administered every 3.5 days, or the single dose divided equally into seven doses and administered daily. Treatment duration was 21 days. The PK/PD index (either *C*_max_/MIC, AUC/MIC, or %T_MIC_) linked to effect was chosen using Akaike information criteria (AIC) (17). Experiments were performed twice.

### Measurement of thioridazine concentration.

Samples from the HFS-MAC central compartment were analyzed for thioridazine content using a qualified liquid chromatography-tandem mass spectrometry method. The full method and assay characteristics have been described in detail in our prior publications (9). There was no modification of the published method.

### Pharmacokinetic and pharmacodynamic modeling.

To calculate intracellular concentrations, the number of THP-1 macrophages in each 1-ml sample was counted using an automated Coulter counter, which also gave the cell volumes in each sample. Since the cells were spun and supernatant removed, suspended, and lysed in 1 ml phosphate-buffered saline (PBS), the total cell volume was calculated by multiplying the number of cells in each sample by the volume of each cell. Thioridazine intracellular and extracellular concentrations next were comodeled in ADAPT 5. First, a one-compartment, two-compartment, and three-compartment model was assumed, and model parameters were generated. The best compartmental model was then chosen using AIC, Bayesian information criteria, and parsimony. Pharmacokinetic parameter estimates in each model were then used to calculate the AUC in each hollow-fiber system. The relationship between total bacterial burden and thioridazine exposure was examined using the inhibitory sigmoid *E*_max_ model, whose parameters include maximal kill (*E*_max_), the EC_50_, bacterial burden in nontreated systems (*E*_con_), and the Hill slope, or *H*.

## RESULTS

The thioridazine MIC for MAC was 25 mg/liter. In mutation frequency studies, no MAC resistant to 3 times the thioridazine MIC was isolated from a total MAC burden of either 7.9 log_10_ CFU, 8.7 log_10_ CFU, 10.9 log_10_ CFU, or 11.7 log_10_ CFU. Time-kill studies conducted over a 7-day period against extracellular MAC in the test tube showed an *E*_max_ of 2.6 log_10_ CFU/ml and an EC_50_ of 33.68 mg/liter (*r*^2^ = 0.946), whereas against intracellular bacilli in 12-well plates the observed *E*_max_ was 3.2 log_10_ CFU/ml with an EC_50_ of 10.53 mg/liter (*r*^2^ = 0.999) for a similar duration of exposure, demonstrating greater potency against intracellular bacilli.

In the pilot HFS-MAC study for thioridazine, we used RPMI broth with 10% FBS, and there was no bacterial killing for any of the 7 doses with peak concentrations of up to 100 mg/liter over 14 days of daily therapy. This implied either high protein binding or thioridazine stickiness to HFS-MAC. MRSA killing assays identified an EC_50_ of 13.72 ± 0.669 with 0% FBS, 21.82 ± 0.806 with 2% FBS, and 30.960 ± 2.138 with 10% FBS. Thus, potency fell 59% with 2% FBS and >90% with 10% FBS. The FBS in our HFS-TB MAC therefore was reduced to 2% FBS for subsequent HFS-MAC studies.

**FIG 1:**
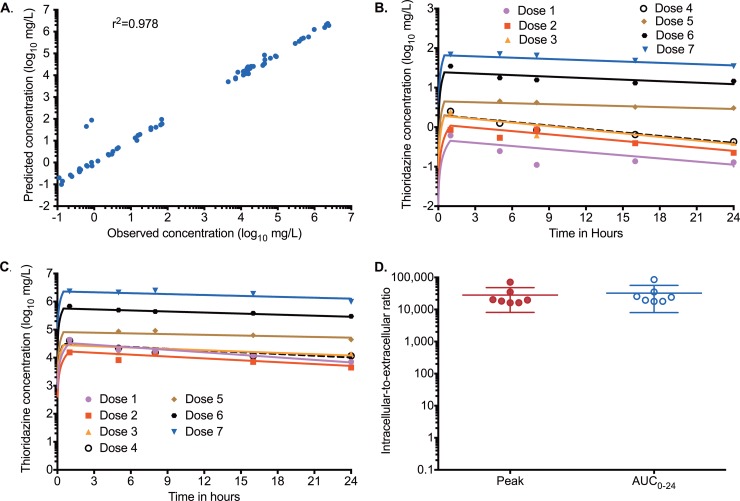
Intracellular and extracellular pharmacokinetics of thioridazine in the HFS-MAC model. (A) Model-predicted concentrations of thioridazine correlated well with observed concentrations. (B) Thioridazine concentration-time profiles achieved extracellularly. (C) Thioridazine concentration-time profiles achieved intracellularly. It should be noted that the log-scale ranges are different, with extracellular concentrations below 2 log_10_ mg/liter and intracellular concentrations that are >6 log_10_ mg/liter. (D) Ratio of intracellular to extracellular thioridazine peak and AUC concentrations.

The thioridazine concentrations assayed at each of the 6 time points in the optimized HFS-MAC were best described by a two-compartment model. The elimination rate constant was 0.042 ± 0.071 h^−1^ and the volume was 6.12 ± 2.45 liters in the extracellular compartment (similar to plasma), while the elimination constant was 0.035 ± 0.06 h^−1^ and the volume 0.16 ± 0.17 liters inside the infected macrophages. The observed concentrations versus model predicted concentrations are shown in Fig. 1A. The concentration-time profiles achieved extracellularly and intracellularly are shown in Fig. 1B and C, and reveal that very high concentrations were achieved intracellularly compared to extracellularly. The ratios of intracellular-to-extracellular peak concentrations and AUC_0–24_ are shown in Fig. 1D and were >20,000-fold.

**FIG 2:**
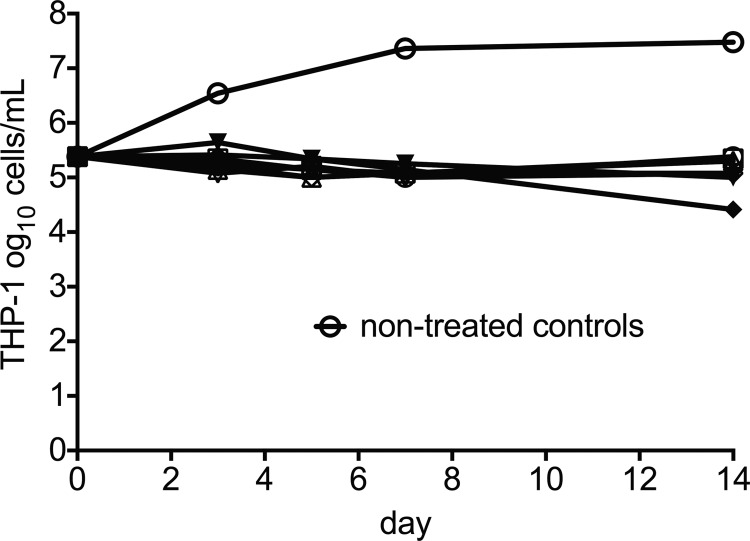
Effect of thioridazine treatment on THP-1 counts. The lowest counts on day 21 were actually due to the second lowest thioridazine concentrations.

**FIG 3:**
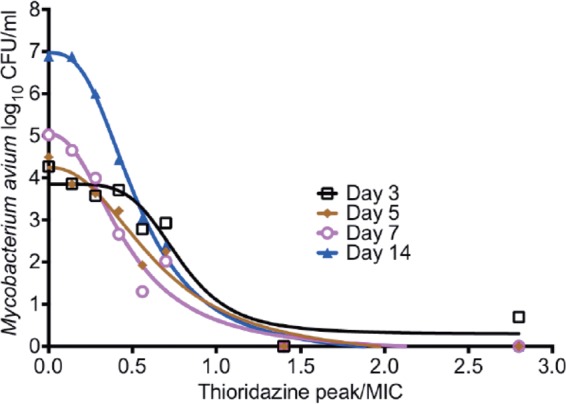
Thioridazine dose-effect study in the HFS-MAC. Shown is the thioridazine microbial kill at different time points during the course of the study, modeled using an inhibitory sigmoid *E*_max_ model for each day.

The effect of these high thioridazine concentrations on THP-1 cells, based on cell counts, is shown in Fig. 2. The macrophages were examined under the microscope on each sampling day and did not show any visible differences from those in non-thioridazine-treated hollow-fiber systems throughout the study. The HFS-MAC bacterial exposure-response results are shown in Fig. 3. The day zero bacterial burden was 5.38 log_10_ CFU/ml. In this case, the inhibitory sigmoid *E*_max_ relationship was expressed as *C*_max_/MIC (see below). The maximal killing (*E*_max_) on day 3 was 3.55 ± 0.59 log_10_ CFU/ml, on day 5 was 4.58 ± 0.75 log_10_ CFU/ml, and on day 7 was 5.20 ± 0.74 log_10_ CFU/ml, which are the highest rates of killing at these time points we have encountered in the HFS-MAC (11, 12). The exposure-effect relationship for bacterial burden on day 14 was described by an inhibitory sigmoid *E*_max_ model with the following parameters: *E*_con_ of 6.96 ± 0.17 log_10_ CFU/ml, *E*_max_ of 7.19 ± 0.30 log_10_ CFU/ml, *H* of 2.75 ± 0.33, and an EC_50_ that was a *C*_max_/MIC of 0.53 ± 0.022 mg/liter (*r*^2^ = 0.996). Based on this, the EC_20_ was a *C*_max_/MIC of 0.32 and the EC_80_ a *C*_max_/MIC of 0.88, which were the exposures used in the dose fractionation studies.

**FIG 4:**
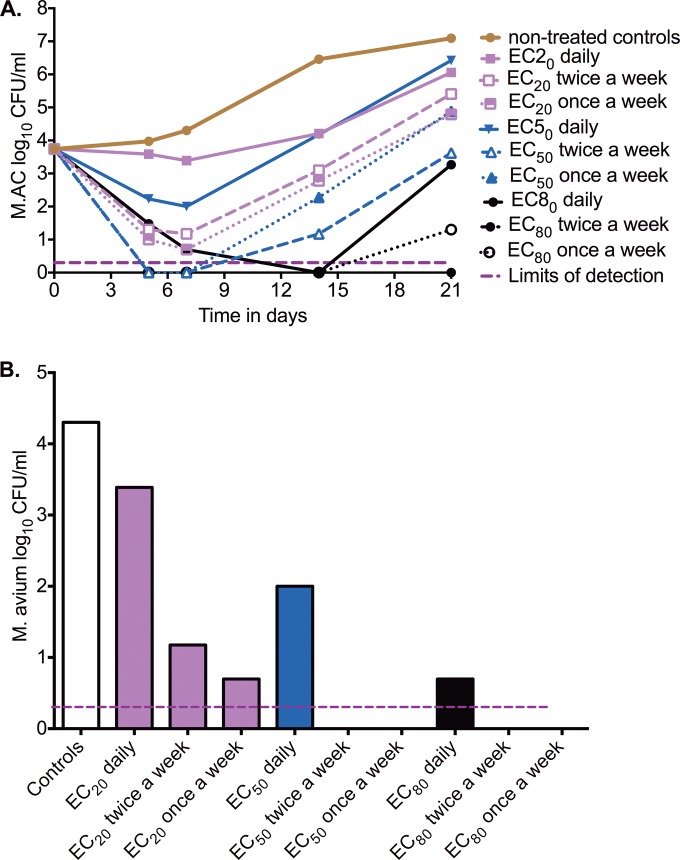
Thioridazine dose scheduling studies in the HFS-MAC. (A) The different thioridazine dosing regimens. There is decreased microbial response beyond day 7 in each dosing regimen. (B) Effect of thioridazine intracellular microbial killing on day 7. The once-daily dosing regimens are associated with the poorest response; for the lowest dose the twice-a-week effect is poorer than that of once-a-week treatment but better than daily therapy. Since the *C*_max_/MIC ratio change in that order while the cumulative AUC/MIC is the same within each dose and the %*T*_MIC_ is in the reverse order, this is an *C*_max_/MIC-driven drug. The purple hatched line indicates limits of detection of the assay. All counts below limits of detection were considered 0.

Dose fractionation study results are shown in Fig. 4. Figure 4A shows that each of the doses administered had considerable microbial killing until day 7. However, after that there was a rebound due to emergence of a drug-resistant subpopulation. The effect of thioridazine on intracellular microbial killing was examined on day 7, at the end of the first dosing interval. Results are shown in Fig. 4B, which shows that the once-a-week regimen was associated with the most microbial killing on day 7 at the lowest dose, and at all doses the daily therapy regimen was associated with the worst microbial effect. Thus, this pattern of microbial killing was consistent with a *C*_max_/MIC linked effect, since for each weekly cumulative AUC/MIC (i.e., either EC_20_, EC_50_, or EC_80_), the once-a-week dose which had the highest *C*_max_/MIC had the best microbial killing. Indeed, the *C*_max_/MIC was also chosen based on the lowest AIC score. Acquired drug resistance, defined as growth at 2× MIC, was observed as early as day 7 in the dose fractionation study. Five of the isolates resistant to 2× MIC were selected and shown to grow in broth supplemented with thioridazine concentrations of 3 times the MIC (i.e., 75 mg/liter). Since the daily regimen always had the worst bacterial burden after day 7 when the total population in the HFS-MAC was being replaced by the thioridazine-resistant subpopulation, suppression of resistance emergence was also inferred to be driven by *C*_max_/MIC.

**FIG 5:**
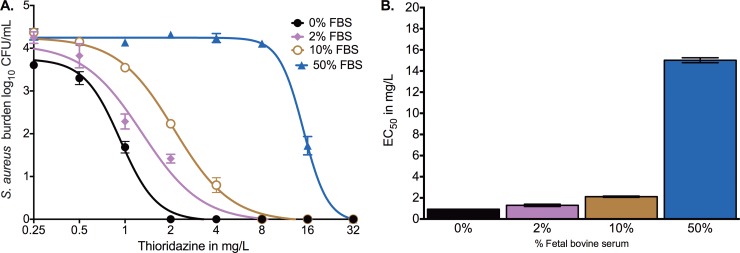
Effect of change in FBS concentration on MRSA killing by thioridazine. (A) Inhibitory sigmoid *E*_max_ comparisons for different percentages of FBS. (B) Change in EC_50_ with percent FBS.

Since studies had been performed in HFS-MAC that used 2% FBS because of the failure of the 10% FBS studies, we wanted to further characterize the effect of protein binding on intracellular microbial killing. We utilized an assay for intracellular MRSA for quick readout, with results shown in Fig. 5. Figure 5A shows inhibitory sigmoid *E*_max_ curves for 0%, 2%, 10%, and 50% FBS. Testing of the null hypothesis that there was no difference in the EC_50_ by percent FBS revealed that the null hypothesis should be rejected and that the EC_50_s were statistically different (*P* < 0.0001). The different EC_50_s are shown in Fig. 5B; the EC_50_ for 50% FBS was 16.4-fold higher than that with 0% FBS. Thus, despite the very high intracellular concentrations, microbial killing could still be constrained by high protein binding.

## DISCUSSION

First, on literature review we found that one study had examined the *in vitro* activity of phenothiazines against MAC (8) and a couple of others that looked at the role of thioridazine as an efflux pump inhibitor for MAC (18, 19), as well as a review article (20), but we found no antimicrobial PK/PD studies of thioridazine and MAC or studies on the intracellular pharmacokinetics of the drug. Here, we show the intracellular pharmacokinetics of thioridazine in response to dynamic extracellular concentrations, which have hitherto never been described before. The hollow-fiber system design, and the repetitive sampling it allows, made this study of intracellular pharmacokinetics possible (21–23). We found that the intracellular pharmacokinetic system differs from the extracellular one by virtue of high thioridazine penetration rates into macrophage and a somewhat lower elimination rate intracellularly. The physiological explanation could be the phenomenon of lysosomal trapping. Daniel and Wojcikowski have shown that under steady-state conditions the highest uptake of thioridazine in tissues coincubated with the drug occurred in adipose tissue, liver, lungs, kidneys, brain, and heart, in that descending order, and attributed it to lysosomal trapping, since this is the same order of lysosomal richness of these tissues (10, 24–31). The same mechanism could be responsible for the high thioridazine concentration in infected macrophages. This suggests that the intracellular pathogen will be exposed to higher concentration-time profiles than extracellularly, a phenomenon also observed with macrolides such as azithromycin and accounting for the remarkable efficacy of the macrolides in the treatment of pneumonia (32).

Second, our studies show that thioridazine is highly protein bound, which limits its effect. We had to alter our experimental design when thioridazine failed to work at high protein concentrations. The central role of protein binding in PK/PD sciences is well known and could explain some of the failures of thioridazine as an effective antimicrobial agent *in vivo* to date (12, 16, 33, 34). When we increased the percentage of FBS, and therefore protein content of the culture media, there was a dramatic increase in EC_50_ for the killing of intracellular MRSA. This suggests that protein binding could still pose a challenge in use of thioridazine as an anti-infective, especially in the case of disseminated MAC.

Third, despite the effect of protein binding, a case can still be made for examination of thioridazine use in chronic pneumonia caused by MAC. While this is an area of considerable controversy, the most comprehensive analysis on the subject found that there is negligible protein binding of any drug in lung epithelial lining fluid in pneumonia (35). This could explain why many highly protein-bound drugs such as rifampin are still highly effective in the treatment of cavitary pneumonias. In addition, thioridazine and other phenothiazines accumulate and concentrate severalfold in the lungs based on animal studies and autopsy studies of poisoning victims (36–39). Thus, it could be that normal doses achieve the high lung concentrations required for the treatment of pulmonary MAC, overcoming any potential drawbacks of protein binding. Thioridazine efficacy was *C*_max_/MIC linked, which was the same PK/PD driver as that in the extracellular tuberculosis model (9). This means that thioridazine could be administered on a more intermittent dosing schedule for pneumonia, thereby potentially limiting dose-related toxicity while maximizing efficacy. However, due to a lack of population pharmacokinetic analysis studies with thioridazine, we could not perform formal computer-aided clinical trial simulations to derive optimal doses for efficacy in patients. Future studies on population pharmacokinetic analysis and identification of thioridazine penetration and protein binding in lungs and epithelial lining fluid in patients similar to that performed for several other high-protein-bound drugs used to treat cavitary pneumonia will be needed to identify optimal doses for efficacy in patients (40–42).

Fourth, in our *in vitro* studies using standard assays to identify the prevalence of resistant mutants, we repeatedly found no resistant mutants with up to ∼12 log_10_ CFU of MAC. This is remarkable, given that resistance was described using a low concentration of 3 times the MIC. This 3× MIC threshold was chosen in order to capture resistance from efflux pump induction, which tends to be low level. On the other hand, resistance emerged quickly in the hollow-fiber system in which the MAC encountered dynamic concentrations of thioridazine. This illustrates several issues. The first is that oscillation of the stressor, as happens in the pharmacokinetic system made possible by the hollow-fiber system, leads to higher mutation rates related to adaptive evolution, while in a constant stressor environment, such as that with static concentrations, mutations are more often deleterious (43–46). Indeed, “the shape, size, variance, and asymmetry of environmental fluctuation have different but predictable effects on evolutionary dynamics” (47). The HFS-MAC and its predecessor, the HFS-TB, give us the ability to precisely shape, size, and vary chemical stressors such as antibiotics and capture the resulting evolutionary adaptation. In the case of MAC and thioridazine, this led to dramatic differences in the emergence of resistance compared to the standard assay.

The Achilles' heel of thioridazine use is its high rates of neurological and cardiac toxicity. The toxicity is mainly driven by one of thioridazine's metabolites, especially thioridazine 5-sulfoxide. This metabolite is highly arrhythmogenic and can lead to heart block (48, 49). Because of the concentration-related toxicity, thioridazine used to be one of the most commonly used drugs for suicide. These factors led to abandonment of thioridazine use in psychiatry. Thus, thioridazine use as an antibiotic could be curtailed for the same reasons. In our hollow-fiber study, we found that high-dose thioridazine use did not lead to death of the THP-1 cells; however, their growth was suppressed (Fig. 2). This is because THP-1 cells are in fact a leukemia cell line from an acute monocytic leukemia patient, and it has been well documented that phenothiazines, even at micromolar concentrations, suppress proliferation of leukemic cells but have no influence on viability of normal lymphocytes (50). Thus, this mechanism is not expected to lead to adverse events. Examination of the possible use of thioridazine in pulmonary MAC will require a balance between several factors. Nevertheless, while we think thioridazine could be effective for the treatment of pulmonary MAC, a more practical use of our findings could be the development of congeners that can achieve the same high intracellular concentrations and target *C*_max_/MIC values but with metabolites that do not have the associated toxicity.

## References

[1] Griffith DE, Aksamit T, Brown-Elliott BA, Catanzaro A, Daley C, Gordin F, Holland SM, Horsburgh R, Huitt G, Iademarco MF, Iseman M, Olivier K, Ruoss S, von Reyn CF, Wallace RJ Jr, Winthrop K 2007 An official ATS/IDSA statement: diagnosis, treatment, and prevention of nontuberculous mycobacterial diseases. Am J Respir Crit Care Med 175:367–416. doi:10.1164/rccm.200604-571ST. 17277290

[2] 1997 Diagnosis and treatment of disease caused by nontuberculous mycobacteria. Am J Respir Crit Care Med 156:S1–25. doi:10.1164/ajrccm.156.2.atsstatement. 9279284

[3] Hibiya K, Shigeto E, Iida K, Kaibai M, Higa F, Tateyama M, Fujita J 2012 Distribution of mycobacterial antigen based on differences of histological characteristics in pulmonary Mycobacterium avium infectious diseases–consideration of the extent of surgical resection from the pathological standpoint. Pathol Res Pract 208:53–58. doi:10.1016/j.prp.2011.10.001. 22177729

[4] Hafner R, Inderlied CB, Peterson DM, Wright DJ, Standiford HC, Drusano G, Muth K 1999 Correlation of quantitative bone marrow and blood cultures in AIDS patients with disseminated Mycobacterium avium complex infection. J Infect Dis 180:438–447. doi:10.1086/314865. 10395860

[5] Horsburgh CR Jr, Metchock B, Gordon SM, Havlik JA Jr, McGowan JE Jr, Thompson SE III 1994 Predictors of survival in patients with AIDS and disseminated Mycobacterium avium complex disease. J Infect Dis 170:573–577. doi:10.1093/infdis/170.3.573. 8077714

[6] Amaral L, Martins M, Viveiros M 2007 Enhanced killing of intracellular multidrug-resistant Mycobacterium tuberculosis by compounds that affect the activity of efflux pumps. J Antimicrob Chemother 59:1237–1246. doi:10.1093/jac/dkl500. 17218448

[7] Silva PE, Bigi F, Santangelo MP, Romano MI, Martin C, Cataldi A, Ainsa JA 2001 Characterization of P55, a multidrug efflux pump in Mycobacterium bovis and Mycobacterium tuberculosis. Antimicrob Agents Chemother 45:800–804. doi:10.1128/AAC.45.3.800-804.2001. 11181364 PMC90377

[8] Viveiros M, Martins M, Couto I, Kristiansen JE, Molnar J, Amaral L 2005 The in vitro activity of phenothiazines against Mycobacterium avium: potential of thioridazine for therapy of the co-infected AIDS patient. In Vivo 19:733–736. 15999542

[9] Musuka S, Srivastava S, Siyambalapitiyage Dona CW, Meek C, Leff R, Pasipanodya J, Gumbo T 2013 Thioridazine pharmacokinetic-pharmacodynamic parameters “wobble” during treatment of tuberculosis: a theoretical basis for shorter-duration curative monotherapy with congeners. Antimicrob Agents Chemother 57:5870–5877. doi:10.1128/AAC.00829-13. 24041886 PMC3837896

[10] Daniel WA, Wojcikowski J 1999 The role of lysosomes in the cellular distribution of thioridazine and potential drug interactions. Toxicol Appl Pharmacol 158:115–124. doi:10.1006/taap.1999.8688. 10406926

[11] Deshpande D, Srivastava S, Meek C, Leff R, Gumbo T 2010 Ethambutol optimal clinical dose and susceptibility breakpoint identification by use of a novel pharmacokinetic-pharmacodynamic model of disseminated intracellular Mycobacterium avium. Antimicrob Agents Chemother 54:1728–1733. doi:10.1128/AAC.01355-09. 20231389 PMC2863641

[12] Deshpande D, Srivastava S, Meek C, Leff R, Hall GS, Gumbo T 2010 Moxifloxacin pharmacokinetics/pharmacodynamics and optimal dose and susceptibility breakpoint identification for treatment of disseminated Mycobacterium avium infection. Antimicrob Agents Chemother 54:2534–2539. doi:10.1128/AAC.01761-09. 20385862 PMC2876403

[13] Deshpande D, Gumbo T 2011 Pharmacokinetic/pharmacodynamic-based treatment of disseminated Mycobacterium avium. Future Microbiol 6:433–439. doi:10.2217/fmb.11.25. 21526944

[14] Schmalstieg AM, Srivastava S, Belkaya S, Deshpande D, Meek C, Leff R, van Oers NS, Gumbo T 2012 The antibiotic resistance arrow of time: efflux pump induction is a general first step in the evolution of mycobacterial drug resistance. Antimicrob Agents Chemother 56:4806–4815. doi:10.1128/AAC.05546-11. 22751536 PMC3421847

[15] Muusze RG, Vanderheeren FA 1977 Plasma levels and half lives of thioridazine and some of its metabolites. II. Low doses in older psychiatric patients. Eur J Clin Pharmacol 11:141–147. 837967 10.1007/BF00562906

[16] Gumbo T, Angulo-Barturen I, Ferrer-Bazaga S 2015 Pharmacokinetic-pharmacodynamic and dose-response relationships of antituberculosis drugs: recommendations and standards for industry and academia. J Infect Dis 211(Suppl 3):S96–S106. doi:10.1093/infdis/jiu610. 26009618

[17] Akaike H 1974 A new look at the statistical model identification. IEEE Trans Automated Control 19:716–723. doi:10.1109/TAC.1974.1100705.

[18] Rodrigues L, Sampaio D, Couto I, Machado D, Kern WV, Amaral L, Viveiros M 2009 The role of efflux pumps in macrolide resistance in Mycobacterium avium complex. Int J Antimicrob Agents 34:529–533. doi:10.1016/j.ijantimicag.2009.07.010. 19740629

[19] Rodrigues L, Wagner D, Viveiros M, Sampaio D, Couto I, Vavra M, Kern WV, Amaral L 2008 Thioridazine and chlorpromazine inhibition of ethidium bromide efflux in Mycobacterium avium and Mycobacterium smegmatis. J Antimicrob Chemother 61:1076–1082. doi:10.1093/jac/dkn070. 18310137

[20] van Ingen J 2011 The broad-spectrum antimycobacterial activities of phenothiazines, InVitro: somewhere in all of this there may be patentable potentials. Recent Pat Antiinfect Drug Discov 6:104–109. doi:10.2174/157489111796064623. 21517740

[21] Gumbo T, Pasipanodya JG, Nuermberger E, Romero K, Hanna D 2015 Correlations between the hollow fiber model of tuberculosis and therapeutic events in tuberculosis patients: learn and confirm. Clin Infect Dis 61(Suppl 1):S18–S24. doi:10.1093/cid/civ426. 26224768

[22] Pasipanodya JG, Nuermberger E, Romero K, Hanna D, Gumbo T 2015 Systematic analysis of hollow fiber model of tuberculosis experiments. Clin Infect Dis 61(Suppl 1):S10–S17. doi:10.1093/cid/civ425. 26224767

[23] Gumbo T, Pasipanodya JG, Romero K, Hanna D, Nuermberger E 2015 Forecasting accuracy of the hollow fiber model of tuberculosis for clinical therapeutic outcomes. Clin Infect Dis 61(Suppl 1):S25–S31. doi:10.1093/cid/civ427. 26224769

[24] Bickel MH, Steele JW 1974 Binding of basic and acidic drugs to rat tissue subcellular fractions. Chem Biol Interact 8:151–162. doi:10.1016/0009-2797(74)90037-4. 4823117

[25] Francesco CD, Bickel MH 1977 Membrane lipids as intracellular binders of chlorpromazine and related drugs. Chem Biol Interact 16:335–346. doi:10.1016/0009-2797(77)90113-2. 862134

[26] Romer J, Bickel MH 1979 Interactions of chlorpromazine and imipramine with artificial membranes investigated by equilibrium dialysis, dual-wavelength photometry, and fluorimetry. Biochem Pharmacol 28:799–805. doi:10.1016/0006-2952(79)90361-7. 454478

[27] MacIntyre AC, Cutler DJ 1988 Role of lysosomes in hepatic accumulation of chloroquine. J Pharm Sci 77:196–199. doi:10.1002/jps.2600770303. 3373422

[28] MacIntyre AC, Cutler DJ 1988 The potential role of lysosomes in tissue distribution of weak bases. Biopharm Drug Dispos 9:513–526. doi:10.1002/bod.2510090602. 3067757

[29] Miles PR, Bowman L, Castranova V 1985 Incorporation of [3H]palmitate and [14C]choline into disaturated phosphatidylcholines in rat alveolar macrophages. Biochim Biophys Acta 833:342–350. doi:10.1016/0005-2760(85)90208-5. 3970960

[30] Haagsman HP, van Golde LM 1991 Synthesis and assembly of lung surfactant. Annu Rev Physiol 53:441–464. doi:10.1146/annurev.ph.53.030191.002301. 2042968

[31] Williams MC, Hawgood S, Hamilton RL 1991 Changes in lipid structure produced by surfactant proteins SP-A, SP-B, and SP-C. Am J Respir Cell Mol Biol 5:41–50. doi:10.1165/ajrcmb/5.1.41. 1878252

[32] Danesi R, Lupetti A, Barbara C, Ghelardi E, Chella A, Malizia T, Senesi S, Angeletti CA, Del Tacca M, Campa M 2003 Comparative distribution of azithromycin in lung tissue of patients given oral daily doses of 500 and 1000 mg. J Antimicrob Chemother 51:939–945. doi:10.1093/jac/dkg138. 12654753

[33] Craig WA, Kunin CM 1976 Significance of serum protein and tissue binding of antimicrobial agents. Annu Rev Med 27:287–300. doi:10.1146/annurev.me.27.020176.001443. 180874

[34] Zeitlinger MA, Derendorf H, Mouton JW, Cars O, Craig WA, Andes D, Theuretzbacher U 2011 Protein binding: do we ever learn? Antimicrob Agents Chemother 55:3067–3074. doi:10.1128/AAC.01433-10. 21537013 PMC3122431

[35] Kiem S, Schentag JJ 2008 Interpretation of antibiotic concentration ratios measured in epithelial lining fluid. Antimicrob Agents Chemother 52:24–36. doi:10.1128/AAC.00133-06. 17846133 PMC2223903

[36] Dutta NK, Pinn ML, Karakousis PC 2014 Reduced emergence of isoniazid resistance with concurrent use of thioridazine against acute murine tuberculosis. Antimicrob Agents Chemother 58:4048–4053. doi:10.1128/AAC.02981-14. 24798290 PMC4068531

[37] Dinovo EC, Bost RO, Sunshine I, Gottschalk LA 1978 Distribution of thioridazine and its metabolites in human tissues and fluids obtained postmortem. Clin Chem 24:1828–1830. 699294

[38] Hackman CR, Rosengard S, Vapaatalo H 1970 Tissue distribution of chlorpromazine studied by microautoradiography. Eur J Pharmacol 9:59–66. doi:10.1016/0014-2999(70)90321-3. 5434294

[39] Forrest FM, Forrest IS, Roizin L 1963 Clinical, biochemical and post mortem studies on a patient treated with chlorpromazine. Agressologie 4:259–265. 13958777

[40] Rodvold KA, George JM, Yoo L 2011 Penetration of anti-infective agents into pulmonary epithelial lining fluid: focus on antibacterial agents. Clin Pharmacokinet 50:637–664. doi:10.2165/11594090-000000000-00000. 21895037

[41] Clewe O, Goutelle S, Conte JE, Simonsson US 2015 A pharmacometric pulmonary model predicting the extent and rate of distribution from plasma to epithelial lining fluid and alveolar cells-using rifampicin as an example. Eur J Clin Pharmacol 71:313–319. doi:10.1007/s00228-014-1798-3. 25620089 PMC4333237

[42] Walsh TJ, Goutelle S, Jelliffe RW, Golden JA, Little EA, DeVoe C, Mickiene D, Hayes M, Conte JE Jr 2010 Intrapulmonary pharmacokinetics and pharmacodynamics of micafungin in adult lung transplant patients. Antimicrob Agents Chemother 54:3451–3459. doi:10.1128/AAC.01647-09. 20439610 PMC2916355

[43] Taddei F, Radman M, Maynard-Smith J, Toupance B, Gouyon PH, Godelle B 1997 Role of mutator alleles in adaptive evolution. Nature 387:700–702. doi:10.1038/42696. 9192893

[44] Ishii K, Matsuda H, Iwasa Y, Sasaki A 1989 Evolutionarily stable mutation rate in a periodically changing environment. Genetics 121:163–174. 17246489 10.1093/genetics/121.1.163 PMC1203599

[45] Feldman MW, Liberman U 1986 An evolutionary reduction principle for genetic modifiers. Proc Natl Acad Sci U S A 83:4824–4827. doi:10.1073/pnas.83.13.4824. 3460074 PMC323834

[46] Liberman U, Feldman MW 1986 Modifiers of mutation rate: a general reduction principle. Theor Popul Biol 30:125–142. doi:10.1016/0040-5809(86)90028-6. 3750215

[47] Carja O, Liberman U, Feldman MW 2014 Evolution in changing environments: modifiers of mutation, recombination, and migration. Proc Natl Acad Sci U S A 111:17935–17940. doi:10.1073/pnas.1417664111. 25427794 PMC4273399

[48] Hale PW Jr, Poklis A 1986 Cardiotoxicity of thioridazine and two stereoisomeric forms of thioridazine 5-sulfoxide in the isolated perfused rat heart. Toxicol Appl Pharmacol 86:44–55. doi:10.1016/0041-008X(86)90398-4. 3764935

[49] Hale P, Poklis A 1996 Thioridazine cardiotoxicity. J Toxicol Clin Toxicol 34:127–230. doi:10.3109/15563659609020247. 8632504

[50] Zhelev Z, Ohba H, Bakalova R, Hadjimitova V, Ishikawa M, Shinohara Y, Baba Y 2004 Phenothiazines suppress proliferation and induce apoptosis in cultured leukemic cells without any influence on the viability of normal lymphocytes. Phenothiazines and leukemia. Cancer Chemother Pharmacol 53:267–275. doi:10.1007/s00280-003-0738-1. 14663628

